# Transvenous Lead Extraction in a Real-World Cohort: Clinical Characteristics, Outcomes, and Performance of the SAFeTY Score

**DOI:** 10.3390/jcm15145745

**Published:** 2026-07-22

**Authors:** Mark Racman, Aida Duric, Jan Kafol, Jan Prevolnik, Jus Ksela

**Affiliations:** 1 Department of Cardiovascular Surgery, University Medical Centre Ljubljana, Zaloska 7, 1000 Ljubljana, Sloveniaaida.duric@kclj.si (A.D.); jan.prevo@hotmail.com (J.P.); 2 Faculty of Medicine, University of Ljubljana, 1000 Ljubljana, Slovenia; 3 Department of Vascular Diseases, Division of Internal Medicine, University Medical Centre Ljubljana, 1000 Ljubljana, Slovenia

**Keywords:** transvenous lead extraction, cardiovascular implantable electronic devices, SAFeTY score, risk stratification, mechanical rotational sheath

## Abstract

**Background/Objectives**: The increasing use of cardiovascular implantable electronic devices has increased the need for transvenous lead extraction (TLE), which carries a relevant complication risk. The SAFeTY score was developed to predict procedure-related major complications. We evaluated TLE outcomes, explored associations between the SAFeTY score and retrospective endpoints, and compared conventional extraction (CE) with mechanical rotational sheath-assisted extraction (MRSE). **Methods**: This retrospective study included consecutive patients undergoing TLE at a tertiary center between 2015 and 2025. The SAFeTY score was calculated in patients undergoing MRSE and examined in relation to procedure-related and all-cause in-hospital mortality, significant post-procedural hemoglobin decrease, and a composite endpoint. **Results**: Among 314 patients, 193 (61.5%) underwent MRSE. These patients had longer cumulative lead dwell time and more previous procedures, indicating greater procedural complexity. Overall procedural success was 95.9%, procedure-related mortality was 0.3%, and all-cause in-hospital mortality was 4.8%. Exploratory unadjusted comparisons showed no significant differences between MRSE and CE in procedural success or mortality. In the MRSE group, 17.1% had a post-procedural hemoglobin decrease > 30 g/L. The SAFeTY score was not associated with procedure-related or all-cause mortality but was associated with the composite endpoint (odds ratio 1.12 per point, *p* = 0.025), with modest discrimination (area under the receiver operating characteristic curve [AUC] 0.59). **Conclusions**: TLE achieved high procedural success and very low procedure-related mortality. Because MRSE was non-randomly selected for more complex procedures, the study cannot establish equivalence or an independent effect of extraction strategy. Associations between the SAFeTY score and alternative retrospective endpoints should be considered exploratory and do not constitute validation for its original purpose.

## 1. Introduction

The use of cardiovascular implantable electronic devices (CIEDs) has expanded substantially over recent decades, driven by broader indications and an aging population [1]. This growth has been accompanied by a parallel rise in device-related complications, including infection, lead failure, and the need for system revision or upgrade, all of which frequently require lead removal. Consequently, transvenous lead extraction (TLE) has become an essential component of contemporary CIED management and is recommended as the first-line strategy for complete system removal in most clinical scenarios, offering a minimally invasive and effective alternative to surgical extraction [2,3].

Despite its central role, TLE remains a technically complex procedure associated with a small but clinically relevant risk of major complications, including cardiac or vascular injury, tamponade, and procedure-related death [4,5]. Contemporary large registries report high procedural success but major complication rates of approximately 1–3% [4,6,7,8]. Accordingly, accurate risk stratification is essential to guide procedural planning, including operator expertise, availability of surgical backup, and perioperative management [5,9]. Several predictors of adverse outcomes have been consistently identified, including prolonged lead dwell time, prior procedures, and patient-related factors [10,11]. While structured risk models such as the SAFeTY score have been proposed, their validation in independent real-world cohorts remains limited [12,13].

Therefore, the present study aimed to characterize a real-world cohort undergoing TLE over a 10-year period and to describe the use of conventional extraction (CE) and mechanical rotational sheath-assisted extraction (MRSE) in routine clinical practice. We also evaluated procedural outcomes in the overall cohort and explored associations between the SAFeTY score and retrospectively available adverse-event endpoints [12]. Comparisons between CE and MRSE were exploratory and were intended to describe patient selection, procedural complexity, and observed outcomes rather than to assess comparative effectiveness.

## 2. Materials and Methods

### 2.1. Study Population, Lead Extraction Procedure, and Data Collection

This retrospective observational study included consecutive patients undergoing TLE at the Department of Cardiovascular Surgery, University Medical Centre Ljubljana between January 2015 and October 2025. No additional exclusion criteria were applied. Indications for extraction comprised device-related infection (pocket infection or endocarditis), lead malfunction, system upgrade, or other clinically indicated reasons.

Lead extraction procedures were performed in an operating theatre under general anesthesia and fluoroscopic guidance. Transesophageal echocardiography was available and used selectively when clinically indicated. A standardized stepwise extraction approach was used, as previously described by Ksela et al. [14]. Extraction was initially attempted using conventional techniques, including manual traction, locking stylets, and additional non-powered mechanical support when required. The extraction strategy was not randomly assigned. MRSE was introduced when conventional measures did not permit safe progression or lead removal because of resistant adhesions or other technical difficulties. The decision to escalate to MRSE was made intra-procedurally by the operating team according to technical requirements. Patients were retrospectively classified in the MRSE group when a mechanical rotational sheath was used at any stage of the procedure and in the CE group when extraction was completed without rotational sheath assistance. Consequently, the MRSE group represented procedures of greater technical complexity rather than a prospectively allocated treatment group. MRSE was defined as extraction involving a mechanical rotational dilator sheath system (Cook Medical, Bloomington, IN, USA), whereas CE included manual traction, locking stylets, and other conventional non-powered techniques without the use of a dedicated rotational sheath. Adjunctive techniques, including femoral snaring, were used when necessary to achieve complete extraction.

Baseline demographic, clinical, and device-related characteristics were collected at hospital admission. Procedural details were recorded intraoperatively, and patients were monitored postoperatively, with early complications assessed during hospitalization.

Procedural success was defined as complete removal of all targeted leads and lead material without the occurrence of major adverse events. Procedural failure was defined as inability to achieve complete procedural success, including but not limited to: (1) death during the procedure, (2) retention of a significant portion of lead material (e.g., >4 cm), (3) major vascular complications such as superior vena cava (SVC) injury or thrombosis, or (4) other procedure-related complications preventing successful extraction.

The study was conducted in accordance with the Declaration of Helsinki and approved by the Slovenian Medical Ethics Committee (approval number: 0120-251/2020). All patients had previously provided informed consent for the use of anonymized clinical data for research purposes.

### 2.2. SAFeTY Score and Endpoint Definition

The SAFeTY TLE score, as previously described by Jacheć et al., was calculated for patients undergoing MRSE [12]. The score incorporates clinical and procedural variables associated with major complications during TLE, including the sum of lead dwell times, anemia, female sex, number of prior procedures, and younger age at first implantation. It was originally developed to predict the risk of procedure-related major complications, such as cardiac or vascular injury, hemopericardium, hemothorax, stroke, and procedure-related death. Based on the total score, patients are stratified into four risk categories: low risk (0–4 points), intermediate risk (4.1–10 points), high risk (10.1–16 points), and very high risk (>16 points), corresponding to progressively increasing probabilities of major complications [12].

Due to the retrospective nature of our dataset, detailed adjudication of procedure-related major complications as defined in the original SAFeTY study was not available. Therefore, we focused on clinically relevant and reliably captured endpoints, including all-cause in-hospital mortality and procedure-related mortality. Additional endpoints included post-procedural hemoglobin drop, defined as the difference between pre-procedural hemoglobin and the lowest value recorded during hospitalization. A significant post-procedural hemoglobin decrease was operationally defined as a reduction of >30 g/L from the pre-procedural value to the lowest value recorded during hospitalization. This laboratory endpoint was not considered equivalent to clinically adjudicated bleeding because hemoglobin changes may also reflect hemodilution, fluid administration, repeated blood sampling, or other non-bleeding causes. Standardized bleeding classifications could not be applied because associated clinical bleeding events were not systematically captured in the retrospective dataset [15,16]. A composite exploratory endpoint was defined as all-cause in-hospital death or a significant post-procedural hemoglobin decrease of >30 g/L. Accordingly, this analysis was exploratory and was not intended as an external validation of the SAFeTY score for its original purpose. Instead, we assessed its association with alternative retrospectively available endpoints.

### 2.3. Statistical Methods

Data were collected using Microsoft Excel 365 (Microsoft Corporation, Redmond, WA, USA) and analyzed in R version 4.4.1 (R Foundation for Statistical Computing, Vienna, Austria). Continuous variables are presented as mean ± standard deviation (SD), while categorical variables are reported as counts and percentages. Normality of continuous variables was assessed visually and using the Shapiro–Wilk test. Comparisons between groups (MRSE vs. CE) were performed using Student’s *t*-test or the Mann–Whitney U test for continuous variables, as appropriate. Categorical variables were compared using the χ^2^ test or Fisher’s exact test when expected cell counts were low, with Monte Carlo simulation applied when necessary. Because extraction strategy was selected according to intra-procedural technical requirements rather than by random allocation, comparisons between MRSE and CE were considered exploratory. Non-significant differences were not interpreted as evidence of equivalence between the two strategies. A comprehensive adjusted comparison was limited by the small number of outcome events and incomplete retrospective availability of several potentially relevant confounders.

Exploratory logistic regression analysis was used to evaluate predictors of all-cause in-hospital mortality in the overall cohort. For analyses of the SAFeTY score, only univariate logistic regression models were performed to assess associations with death, significant post-procedural hemoglobin decrease, and the composite endpoint, given that several covariates are incorporated within the score itself. Discriminative performance of the SAFeTY score was assessed using receiver operating characteristic (ROC) curve analysis, with calculation of the area under the curve (AUC). Event rates across SAFeTY risk categories were compared using χ^2^ tests.

All statistical tests were two-tailed, with a significance level set at *p* < 0.05. Missing data were handled by complete case analysis, and no imputation was performed.

## 3. Results

### 3.1. Clinical Characteristics and Extraction Strategy

**Figure 1:**
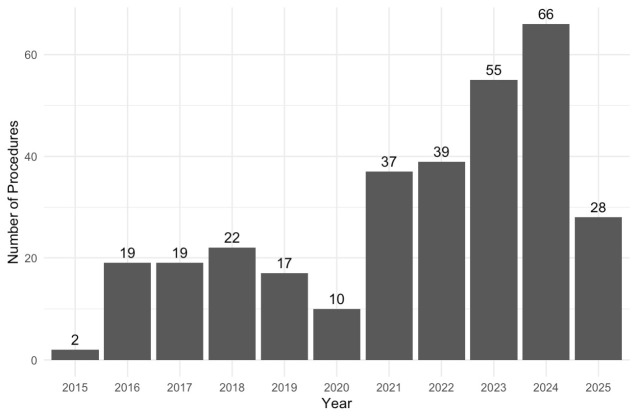
Number of lead extraction procedures performed annually from January 2015 to October 2025.

Between 2015 and October 2025, a total of 314 patients underwent TLE at our center, with a steadily increasing procedural volume over time (Figure 1). Of these, 193 (61.5%) procedures were performed using MRSE (Cook Medical, USA), while the remaining procedures were performed using CE techniques.

The mean age of the overall cohort was 64.8 ± 17.8 years, and 76.4% were male. The most common indication for extraction was pocket infection (36.9%), followed by lead endocarditis (25.5%) and device malfunction (23.9%). Overall, 62.4% of patients underwent extraction due to infection (pocket infection or lead endocarditis). Baseline characteristics of the study population, stratified by extraction method, are summarized in Table 1. The most commonly extracted device type was pacemaker (63.4%), followed by implantable cardioverter-defibrillator (22.6%) and cardiac resynchronization therapy devices (14.0%).

Across the entire cohort, the most frequently documented pathogens were *Staphylococcus aureus* (43/314, 13.7%) and coagulase-negative staphylococci (41/314, 13.1%). Among the 196 patients with infectious indications, these corresponded to 21.9% and 20.9%, respectively. The overall distribution of microbiological categories did not differ between MRSE and CE groups (*p* = 0.146; Table 1).

As shown in Table 1, patients undergoing MRSE system had significantly longer lead dwell time (*p* < 0.001) and a higher number of prior procedures (*p* = 0.009), reflecting increased procedural complexity. No significant differences were observed between groups with respect to age, sex, indication for extraction, device type, or infectious etiology.

### 3.2. Procedural Outcomes

Overall procedural success was achieved in 301 of 314 patients (95.9%). In the CE group, procedural success was achieved in 119 of 121 procedures (98.3%), with 2 failures (1.7%), both due to retention of lead material exceeding 4 cm. In the MRSE group, procedural success was achieved in 182 of 193 procedures (94.3%), with 11 failures (5.7%), including 8 cases of retained lead fragments, 2 cases of superior vena cava thrombosis, and 1 procedure-related death. Procedural success was 98.3% in the CE group and 94.3% in the MRSE group; this difference did not reach statistical significance in the exploratory unadjusted comparison (*p* = 0.09).

Procedure-related mortality occurred in 1 of 314 patients (0.3%). The event occurred in the MRSE group, whereas no procedure-related deaths occurred in the CE group; because only one event was observed, a meaningful adjusted comparison of procedure-related mortality was not possible. Crude all-cause in-hospital mortality was 5.0% in the CE group and 4.7% in the MRSE group (*p* = 1.000). These comparisons were exploratory and unadjusted and should not be interpreted as evidence of equivalent mortality risk. In exploratory regression analysis, increasing age was associated with all-cause in-hospital mortality, whereas extraction method was not statistically associated with the outcome. In the MRSE cohort, the mean post-procedural hemoglobin decrease was 10.85 ± 12.16 g/L, and 17.1% of patients had a decrease of >30 g/L. Two of these patients required red blood cell transfusion. No other clinically associated bleeding events were recorded in the available dataset.

### 3.3. Association Between the SAFeTY Score and Adverse Events

**Figure 2:**
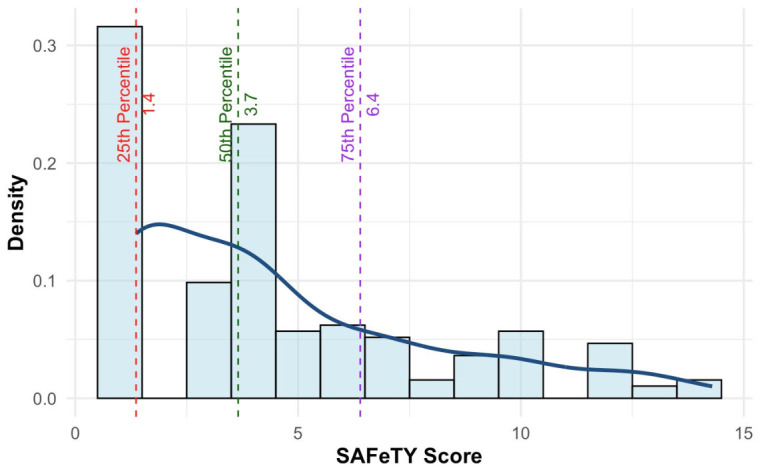
Distribution of SAFeTY score in the mechanical rotational sheath-assisted extraction cohort. Histogram with overlaid kernel density curve illustrating the distribution of SAFeTY scores. The smooth line represents a density estimate, providing a continuous view of the score distribution independent of histogram binning. Vertical dashed lines denote the interquartile range and median.

**Figure 3:**
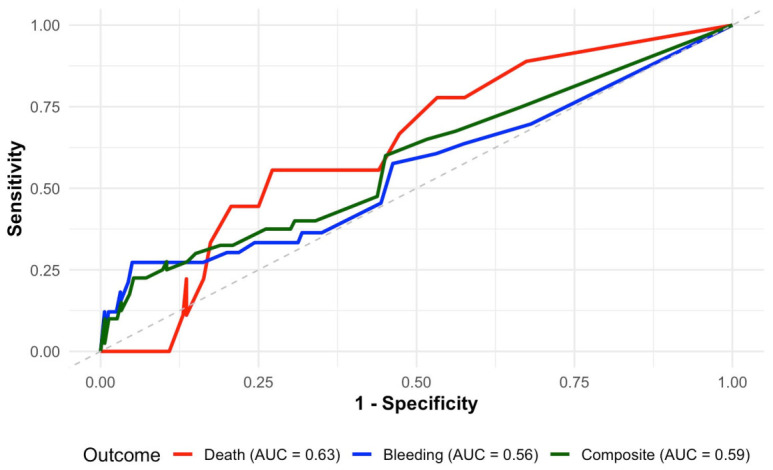
Receiver operating characteristic (ROC) curves for the SAFeTY score in the mechanical rotational sheath-assisted extraction cohort. ROC curves demonstrating the discriminative performance of the SAFeTY score for predicting all-cause death, hemoglobin decrease > 30 g/L, and the composite endpoint.

The SAFeTY score was calculated for patients undergoing MRSE, with a mean value of 4.61 ± 3.42 [12]. The distribution of SAFeTY scores in the MRSE cohort is illustrated in Figure 2. Increasing SAFeTY score was not associated with either procedure-related mortality (*p* = 0.880) or all-cause mortality (*p* = 0.286), but showed a trend toward significant post-procedural hemoglobin decrease (*p* = 0.071) and was significantly associated with the composite endpoint of all-cause death or post-procedural hemoglobin drop (OR 1.12 per point, *p* = 0.025). The discriminative ability of the SAFeTY score was modest, with AUC values of 0.63 for all-cause mortality, 0.56 for significant post-procedural hemoglobin decrease, and 0.59 for the composite endpoint, as shown in Figure 3.

**Figure 4:**
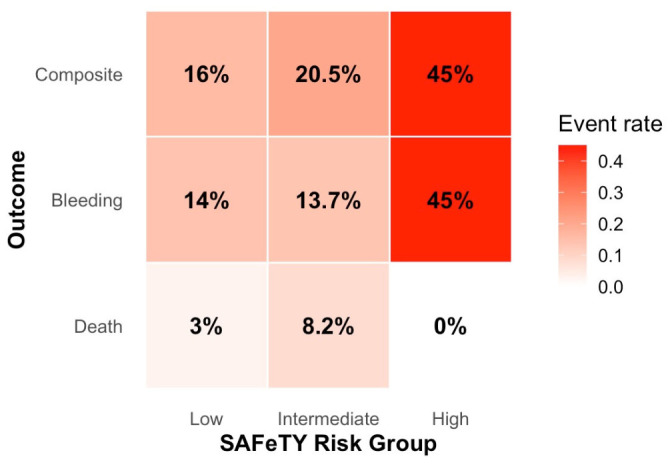
Event rates across SAFeTY risk categories in the mechanical rotational sheath-assisted extraction cohort. Heatmap illustrating the proportion of all-cause death, hemoglobin decrease > 30 g/L, and composite endpoint across SAFeTY risk groups.

When analyzed by SAFeTY risk categories, the majority of patients were classified as low risk (51.8%), followed by intermediate (37.8%) and high risk (10.4%), with no patients meeting criteria for the very high-risk category. As shown in Figure 4, increasing risk category was associated with a clear rise in post-procedural hemoglobin decrease and composite event rates (*p* = 0.002 and *p* = 0.014, respectively), whereas no consistent gradient was observed for procedure-related (*p* = 0.438) and all-cause mortality (*p* = 0.159).

## 4. Discussion

This study provides a comprehensive real-world evaluation of TLE, with a focus on procedural outcomes and risk stratification. We demonstrate that TLE can be performed with high procedural success (>95%) and very low procedure-related mortality, even in a population characterized by substantial clinical complexity. Unadjusted outcome rates did not differ significantly between the extraction groups, although patients requiring MRSE had greater procedural complexity. Because MRSE was selected according to intra-procedural technical requirements rather than randomly assigned, these findings do not establish equivalence or comparative effectiveness between MRSE and CE. Furthermore, the SAFeTY score was associated with selected alternative retrospective endpoints; however, this exploratory analysis did not assess its performance for the original endpoint of systematically adjudicated procedure-related major complications [12].

The demographic profile of our cohort, with a mean age of approximately 65 years and a clear male predominance, is consistent with contemporary TLE registries and large observational studies. Similar age distributions have been reported in other real-world cohorts, reflecting the typical population of patients with long-standing CIEDs [17,18,19]. The predominance of male patients has also been consistently observed and may be partially explained by sex-related differences in CIED implantation rates and underlying cardiovascular disease burden [1,20].

Several measured demographic and clinical characteristics were similar between patients undergoing CE and MRSE, including age, sex, indication for extraction, device type, and infectious etiology. However, patients requiring MRSE had significantly longer lead dwell time and a greater number of previous procedures. This reflects the stepwise procedural strategy used at our center, in which MRSE was introduced when conventional techniques were insufficient because of resistant adhesions or other technical difficulty [21,22]. The two groups should therefore be regarded as procedural pathways of differing complexity rather than directly comparable treatment groups.

Infection—primarily pocket infection and lead-related endocarditis—was the leading indication for TLE. However, reported indications vary across studies, with some cohorts identifying system upgrade or lead dysfunction as the most common reason for extraction [23,24]. The most frequently identified pathogens were *Staphylococcus aureus* and coagulase-negative staphylococci, consistent with the established microbiological profile of CIED-related infections [25]. Although the absolute number of *S. aureus* infections was higher in the MRSE group, their proportional prevalence was nearly identical in the MRSE and CE groups (14.0% vs. 13.2%), and the overall distribution of infectious pathogens did not differ significantly between groups (*p* = 0.146). The higher absolute number therefore primarily reflects the larger size of the MRSE group and does not support a pathogen-specific association with the need for MRSE.

In our cohort, procedural success was 95.9%, and procedure-related mortality was very low (0.3%). These findings were broadly consistent with the outcomes reported in the ELECTRa, PROMET, GALLERY, and original SAFeTY cohorts, as summarized in Table 2 [6,12,21,26]. However, direct comparisons should be interpreted cautiously because the studies differed in patient selection, extraction techniques, definitions of procedural success, lead dwell time, and complication adjudication [4,7,23]. Many of these patients were critically ill at presentation, and their clinical course was likely driven by sepsis, end-organ dysfunction, or underlying disease rather than the extraction procedure itself. Recent evidence suggests that same-day discharge may be feasible after uncomplicated TLE in selected patients, particularly those without device infection and when the procedure is completed early in the day [27]. However, this approach requires careful patient selection and should not be generalized to patients with infection or peri-procedural complications.

In the MRSE cohort, 17.1% of patients experienced a post-procedural hemoglobin decrease of >30 g/L, of whom two required red blood cell transfusion. This laboratory endpoint should not be interpreted as an adjudicated bleeding complication because hemoglobin reduction may also result from non-bleeding causes [4,5,28]. Crude all-cause and procedure-related mortality rates did not differ significantly between groups, despite the greater measured procedural complexity of patients requiring MRSE. However, the absence of a statistically significant difference should not be interpreted as evidence that extraction strategy had no effect on outcome. Non-random technique selection, the low number of events, and incomplete adjustment for potential confounders limit causal interpretation. These findings support the concept that TLE can be performed safely even in high-risk patients, and that the procedure itself is unlikely to be the primary driver of mortality in this setting.

The SAFeTY score showed modest discrimination for the alternative retrospective endpoints evaluated in our cohort and was associated with the composite endpoint and increasing event rates across risk categories. However, these findings should not be interpreted as an external validation of the score for its original purpose. The SAFeTY score was developed to predict systematically adjudicated procedure-related major complications, whereas our retrospective dataset primarily allowed assessment of all-cause in-hospital mortality, procedure-related mortality, significant post-procedural hemoglobin decrease, and a composite of these outcomes. Therefore, the modest AUC values may reflect the mismatch between the available endpoints and the original SAFeTY outcome, incomplete retrospective adjudication, and the low number of events rather than poor performance of the score for its intended purpose. Our findings should consequently be considered exploratory and hypothesis-generating [12].

From a clinical perspective, these findings support a stepwise extraction strategy in which mechanical rotational sheaths are available for procedures complicated by long lead dwell time, repeated previous interventions, or failure of conventional techniques. However, our results should not be used to infer equivalence or superiority of one extraction strategy over another. The SAFeTY score may provide additional information for procedural planning and identification of patients requiring closer monitoring, but it should not be used as a stand-alone decision tool because our analysis did not evaluate its original adjudicated complication endpoint. The marked difference between procedure-related and all-cause in-hospital mortality also highlights the importance of treating infection, sepsis, organ dysfunction, and other comorbidities alongside procedural risk management.

This study has several limitations. Its retrospective design precluded systematic adjudication of the procedure-related major complications used in the original SAFeTY study. Consequently, our analysis assessed associations with alternative endpoints and cannot be considered an external validation of the score [12]. In addition, attribution of mortality to procedural versus non-procedural causes may be subject to misclassification, particularly in critically ill patients. The hemoglobin-based endpoint was not equivalent to a standardized bleeding outcome. Although two patients required transfusion, other clinical bleeding events were not systematically adjudicated, and standardized bleeding classifications could therefore not be retrospectively applied. Hemoglobin data were unavailable for the CE group, preventing direct comparison of significant post-procedural hemoglobin decrease. Additionally, detailed procedural complexity parameters were not systematically captured. The comparison between MRSE and CE was also limited by non-random allocation and confounding by indication. MRSE was generally introduced when conventional extraction was insufficient, meaning that the MRSE group inherently included more technically complex procedures. The small number of outcome events and incomplete availability of several relevant confounders precluded a comprehensive adjusted comparison. Therefore, the between-group findings should be interpreted as descriptive and hypothesis-generating rather than as evidence of equivalence. The single-center design with referral of more complex cases, the relatively low number of events, and the lack of long-term follow-up may further limit generalizability and outcome assessment.

## 5. Conclusions

In this real-world 10-year cohort, transvenous lead extraction was associated with high procedural success and very low procedure-related mortality, despite greater procedural complexity in patients requiring mechanical rotational sheath techniques. All-cause in-hospital mortality was substantially higher than procedure-related mortality, suggesting an important contribution from underlying illness and infection severity. Crude mortality rates did not differ significantly between extraction groups; however, because MRSE was non-randomly selected for more complex procedures and comprehensive adjustment for confounding was not possible, the present study cannot establish equivalence or an independent effect of extraction strategy. The SAFeTY score was associated with selected alternative retrospective endpoints, although discrimination was modest. Because the original procedure-related major complication endpoint was not systematically adjudicated, these findings should not be interpreted as validation of the score or as evidence of poor performance for its intended purpose. These findings highlight the importance of distinguishing procedural safety from patient-related risk and underscore the need for improved and externally validated risk stratification tools in contemporary clinical practice.

## Figures and Tables

**Table 1:** Baseline characteristics according to extraction method.

Variable	CE (n = 121)	MRSE (n = 193)	*p*-Value
**Age (years)**	66.9 ± 15.9	63.5 ± 18.9	0.107
**Female sex**	26 (21.5%)	48 (24.9%)	0.582
**Indication for extraction**			0.080
Skin erosion/exposure	8 (6.6%)	16 (8.3%)	
Device malfunction	21 (17.4%)	54 (28.0%)	
Pocket infection	48 (39.7%)	68 (35.2%)	
Lead endocarditis	32 (26.4%)	48 (24.9%)	
System upgrade	5 (4.1%)	3 (1.6%)	
Cardiac perforation	5 (4.1%)	1 (0.5%)	
Unknown	2 (1.7%)	3 (1.6%)	
**Device type**, n (%)			0.641
Pacemaker	79 (65.3%)	120 (62.2%)	
Implantable cardioverter-defibrillator	24 (19.8%)	47 (24.4%)	
Cardiac resynchronization therapy	18 (14.9%)	26 (13.5%)	
**Infectious status/pathogen**			0.146
No infection/unknown	62 (51.2%)	83 (43.0%)	
*Staphylococcus aureus*	16 (13.2%)	27 (14.0%)	
Coagulase-negative staphylococci	16 (13.2%)	25 (13.0%)	
*Cutibacterium acnes*	6 (5.0%)	27 (14.0%)	
*Streptococcus* spp.	1 (0.8%)	4 (2.1%)	
Gram-negative bacteria	2 (1.7%)	4 (2.1%)	
*Corynebacterium* spp.	1 (0.8%)	2 (1.0%)	
Other rare bacteria	0 (0.0%)	4 (2.1%)	
Polymicrobial infection	17 (14.0%)	17 (8.8%)	
**Lead dwell time (years)**	6.2 ± 9.8 ^a^	13.3 ± 11.6 ^b^	**<0.001**
**Number of prior procedures**	1.27 ± 0.85	1.54 ± 0.86	**0.009**

Continuous variables are presented as mean ± standard deviation and were compared using Student’s *t*-test or the Mann–Whitney U test as appropriate. Categorical variables are presented as counts (percentages) and were compared using the χ^2^ test or Fisher’s exact test, with Monte Carlo simulation applied when expected cell counts were low. MRSE refers to procedures performed using mechanical rotational dilator sheath systems (Cook Medical, USA), while CE refers to procedures performed using conventional extraction techniques, including simple traction or non-powered mechanical methods without dedicated rotational sheath systems. Lead dwell time was calculated as the sum of implantation durations of all extracted leads per patient. The number of prior procedures refers to the total number of previous cardiac implantable electronic device-related interventions, including pacemaker, implantable cardioverter-defibrillator, and cardiac resynchronization therapy procedures. **Abbreviations**: CE—conventional extraction; MRSE—mechanical rotational sheath-assisted extraction. **Missing data**: ^a^ 14 subjects; ^b^ 58 subjects.

**Table 2:** Comparison of the present cohort with major transvenous lead extraction studies.

Study	Design and Setting	Patients, n	Extraction Approach	Infectious Indication	Lead Dwell Time	Procedural Success	Major Procedure-Related Complications	Procedure-Related Mortality
**Present study**	Retrospective, single-centre cohort; 2015–2025	314	Stepwise conventional extraction with escalation to MRSE; MRSE used in 61.5%	62.4%	Cumulative dwell time per patient: CE 6.2 ± 9.8 years; MRSE 13.3 ± 11.6 years	95.9%	Not systematically adjudicated	0.3%
**ELECTRa** [6]	Prospective multicentre European registry; 73 centres in 19 countries	3510	Mixed contemporary techniques; traction alone in 27.3%, non-powered mechanical sheaths in 36.3%, and powered sheaths in 27.1%	52.8%	Mean 6.4 ± 5.4 years; median 5 years (IQR 2–9)	Clinical success 96.7%; complete radiological success 95.7%	1.7%	0.5%
**PROMET** [21]	Retrospective multicentre European study; six centres	2205	Predominantly advanced mechanical techniques; Evolution rotational sheath used in 992 patients	46.0%	Mean 84.7 ± 61.8 months; median 74 months (IQR 41–112)	Clinical success 97.0%; complete extraction per lead 96.5%	1.0%	0.18%
**GALLERY** [26]	Retrospective national registry; 24 German centres	2524	Laser-assisted extraction as the primary approach; additional tools used in 6.65%	63.8%	Median 96 months (IQR 62–141)	Clinical success 97.86%; complete lead removal 94.85%	2.06%	0.55%
**Original SAFeTY cohort** [12]	Single-centre derivation cohort with subsequent prospective validation	2049 derivation; 551 validation	Predominantly non-powered mechanical polypropylene sheaths; powered rotational tools used in <1%	39.8% in the derivation cohort	Cumulative lead dwell time per patient; >16.5 years identified as a major-risk threshold	Complete procedural success 95.0%; clinical success 97.9%	1.81% in derivation cohort; 1.27% in validation cohort	0.39% in derivation cohort; not separately reported for validation cohort

**Abbreviations**: CE, conventional extraction; IQR, interquartile range; MRSE, mechanical rotational sheath-assisted extraction; TLE, transvenous lead extraction. **Note**: Definitions of procedural success, complications, mortality, and lead dwell time differ across studies. The present study and the original SAFeTY cohort report cumulative dwell time per patient; ELECTRa reports extracted-lead dwell time; PROMET reports targeted-lead dwell time; and GALLERY reports the dwell time of the oldest treated lead. These measures are not directly interchangeable. Major procedure-related complications were not systematically adjudicated in the present retrospective cohort.

## Data Availability

Data is available from the corresponding author upon reasonable request.
